# Indirect comparison of etanercept and abatacept efficacy and safety in patients with polyarticular juvenile idiopathic arthritis

**DOI:** 10.1186/1546-0096-12-S1-P132

**Published:** 2014-09-17

**Authors:** OU Loskutova (Konopelko), ES Zholobova, MN Nikolaeva, LA Galstian

**Affiliations:** 1 Department OF Pediatric Rheumatology, First Moscow Medical State University I.M. Sechenov, Moscow, Russian Federation

## Introduction

The use of biological DMARDs have significantly improved the prognosis and prospects for patients with juvenile idiopathic arthritis (JIA). Today the main problem for pediatric rheumatologists when choosing JIA treatment is the absence of comparative controlled studies of efficacy and safety of different biological DMARD.

## Objectives

To compare efficacy and safety of etanercept and abatacept in pediatric patients with polyarticular JIA.

## Methods

The study enrolled 54 pediatric patients with polyarticular JIA, 32 of them received etanercept and 22 received abatacept. The demographic parameters were well matched across treatment groups. The mean age of children was 10.8 ± 3.7, the age at the disease onset was 5.4 ± 3.4, most of the patients were female. Prior to biological DMARD administration, all the subjects received multiple basic immunosuppressants. A total of 68,7 % of subjects in the etanercept arm had disease activity grade II before biological DMARD administration, 31.3% had grade III; 54.6% of subjects in the abatacept arm had disease activity grade I, 31.8% grade II, and 13.6% grade III. American College of Rheumatology “pediatric” criteria (ACR pedi-30, -50, -70, -90), treatment compliance index and index LUNDEX were used to assess efficacy of the study treatment. Biological DMARD efficacy and safety were evaluated at Months 6, 12, 18 and 24 following therapy initiation. The drugs were given at standard doses.

## Results

At least a 50% improvement according to ACR pedi was achieved in 84.3% of etanercept arm subjects and in 71,4% in patients receiving abatacept following 6 months of treatment. Drug-induced clinical and laboratory remission (ACR pedi 90,100) was achieved in 15.6% of subjects in the etanercept arm, and in 9.5% of patients receiving abatacept. After that, biological DMARD efficacy continued to increase. At Month 18, ACR pedi 50 was achieved in 100% and ACR pedi 90 in 31.0% of etanercept subjects; ACR pedi 50 was achieved in 83,3%, and ACR pedi 90 in 33.3% of abatacept subjects. The treatment compliance index at Month 18 was 0,97 in the etanercept arm and 0.8 in the abatacept arm. Index LUNDEX was 0.97 for ACR pedi 50, and 0,3 for ACR pedi 90 in the etanercept arm; in the abatacept arm, it was 0.67 for ACR pedi 50, and 0.27 for ACR pedi 90. At Month 24 all the patients achieved a 50% response according to the ACR pedi criteria. Drug-induced clinical and laboratory remission was achieved in 43.0% of subjects in the etanercept arm, and in 67% of subjects in the abatacept arm. A greater treatment compliance index was obtained in the etanercept arm (0.94 versus 0.6 in the abatacept arm). Thus, when biological DMARD efficacy is compared using index LUNDEX to ACR pedi 50, the best result was obtained with etanercept when compared to abatacept, the values were 0,94 and 0,6, respectively. Index LUNDEX to ACR pedi 90, 100 was 0,4 in both arms. The difference between biological DMARD efficacy was not significant (p<0,05). Adverse drug reactions were more frequent in the abatacept arm (22.7%) than in the etanercept arm (12.5%). Serious adverse reactions were also more frequent with abatacept treatment (9%) than with etanercept (3%; p>0.05).

## Conclusion

Etanercept and abatacept are highly effective drugs for pediatric treatment of polyarticular JIA. Etanercept has a better safety profile than abatacept.

## Disclosure of interest

None declared.

